# Low serum vitamin D is associated with an increased likelihood of acquired premature ejaculation

**DOI:** 10.1590/S1677-5538.IBJU.2018.0887

**Published:** 2019-07-27

**Authors:** Lütfi Canat, Recep Burak Degirmentepe, Hasan Anil Atalay, Suleyman Sami Çakir, Ilter Alkan, Mehmet Gokhan Çulha, Sait Ozbir, Masum Canat

**Affiliations:** 1 Department of Urology, Okmeydani Training and Research Hospital, Istanbul, Turkey; 2 Department of Endocrinology and Metabolism, Şişli Etfal Training and Research Hospital, Istanbul, Turkey

**Keywords:** Premature Ejaculation, Vitamin D, Likelihood Functions

## Abstract

**Purpose:**

To investigate the relationship between 25-hydroxyvitamin D (25 (OH) D) levels and acquired premature ejaculation (PE).

**Materials and Methods:**

A total of 97 patients with acquired PE and 64 healthy men as a control group selected from volunteers without PE attending our Andrology Outpatient Clinic between November 2016 and April 2017 were included the study. All patients were considered to have acquired PE if they fulfilled the criteria of the second Ad Hoc International Society for Sexual Medicine Committee. Premature ejaculation diagnostic tool questionnaires were used to assessment of PE and all participants were instructed to record intravaginal ejaculatory latency time. Vitamin D levels were evaluated in all participants using high performance liquid chromatography method included in the study.

**Results:**

Compared to men without PE, the patients with acquired PE had significantly lower 25 (OH) D levels (12.0 ± 4.5 ng/mL vs. 18.2 ± 7.4 ng/mL, p < 0.001). In the logistic regression analysis, 25 (OH) D was found to be an independent risk factor for acquired PE, with estimated odds ratios (95% CI) of 0.639 (0.460-0.887, p = 0.007) and the area under curve of the ROC curve of 25 (OH) D diagnosing acquired PE was 0.770 (95% CI: 0.695 to 0.844, p < 0.001). The best cut-off value was 16 ng/mL with a sensitivity of 60.9%, specificity of 83.5%, PPV of 70.9%, and NPV of 76.4% to indicate acquired PE.

**Conclusions:**

This study demonstrates that lower vitamin D levels are associated with the acquired PE. The result of our study showed that the role of serum vitamin D levels should be investigate in the etiology of acquired PE. Perhaps supplementation of vitamin D in men with acquired PE will ameliorate the sexual health of these patients.

## INTRODUCTION

Premature ejaculation (PE) is defined as a lack of control on ejaculation and it is the most common sexual dysfunction that may affect 20%– 30% of men (1). Firstly, Godpodinoff defined both types as lifelong and acquired PE (2) and two more types of PE have been suggested as naturel variable PE and premature-like ejaculatory dysfunction by Waldinger and Schweitzer (3, 4). The second Ad Hoc International Society for Sexual Medicine (ISSM) Committee defined PE as a male sexual dysfunction characterized by ‘ejaculation which always or nearly always occurs prior to or within about one minute of vaginal penetration (lifelong PE), or a clinically significant and bothersome reduction in latency time, often to about three minutes or less (acquired PE), the inability to delay ejaculation on all or nearly all vaginal penetration and negative personal consequences (5). The consistent lifelong PE proposed an underlying neurobiological functional disruption, whereas acquired PE is more linked to underlying medical reasons. However, the percentage of patients who have acquired PE of uncertain reason is currently not available (6). Acquired PE is commonly due to anxiety or psychological disturbance (7), erectile dysfunction (ED) (8), hyperthyroidism (9), chronic prostatitis (10), recreational drugs (11), metabolic syndrome (12), and Parkinson's disease (13). Several studies reported that men with acquired PE are more likely to seek medical treatment compared to men with lifelong PE (14, 15). Thus, novel pathophysiological elements may clarify the reasons for this dysfunction and develop new treatment modalities.

Vitamin D is a steroid hormone that is mainly generated in the skin and is converted to vitamin D3 on exposure to sunlight (16). It is a key regulator of bone mineralization and calcium/ phosphorous metabolism. Moreover, expression of the vitamin D receptor in several tissues has been linked to different functions (17). Epidemiological researches have proposed the effect of a pandemic of vitamin D deficiency on non-musculoskeletal functions, including autoimmune disease, infectious disease, neurocognitive and endocrinologic disorders (18).

The biological link between PE and vitamin D deficiency exhibits different mechanisms. Firstly, several animal studies reported that vitamin D deficiency resulted in anxiety-related behaviors (19-21). Anxiety has also been reported as a cause of PE by multiple researches (7). Second, activated vitamin D stimulates the production of nitric oxide (NO) and NO synthases (22). NO and serotonergic system are important at the level of the sympathetic nervous system which can affect the ejaculation (23, 24). Third, when vitamin D increases above its normal range, it binds to the androgen receptor, displacing their native ligands (25). Therefore, several studies have determined that serum androgen levels and vitamin D levels are associated in men (26, 27).

In the present study, we aimed for the first time to evaluate the relationship between serum vitamin D levels and acquired PE.

## MATERIALS AND METHODS

This single-center, prospective, observational study was performed according to the Declaration of Helsinki. The study protocol was approved by the institutional review board of our institution and written informed consent was obtained from all subjects. A total of 161 consecutive men who presented with acquired PE (97 patients) or control group selected from volunteers without PE (64 patients) from November 2016 to April 2017 were included in the study. The participants were included if they were between the ages of 20 and 60 years, were in a stable relationship with a single sexually active partner within the last 6 months and had acquired PE for at least 6 months duration. Exclusion criteria included patients with lifelong PE, the history of psychiatric or neurological disorders, pelvic/perineal trauma, liver or renal failures, urinary tract infection, chronic prostatitis, anatomical abnormalities, and chronic diseases which may affect the sexual functions. The participants who were taking selective serotonin reuptake inhibitors, alpha-blockers, phosphodiesterase inhibitors, anticholinergics, antipsychotics, and vitamin D supplementation were also excluded from the study.

All patients were considered to have acquired PE if they fulfilled the criteria of the second Ad Hoc ISSM Committee (5). All patients were also assessed by the validated five-item Turkish version of Premature Ejaculation Diagnostic Tool (PEDT) (28). To evaluate the erectile function, International Index of Erectile Function-5 (IIEF-5), a validated 15-item self-administered questionnaire that was validated in Turkish was used (29, 30). All participants were instructed to record intravaginal ejaculatory latency time (IELT) using stopwatch which was held by the partner. The Beck Depression Inventory (BDI) form which was validated in Turkish was used for psychological evaluation (31, 32). The body mass index (BMI), smoking history, alcohol consumption, partner age, frequency of intercourse, and associated comorbidities were recorded by face-to-face interview.

Vitamin D and total testosterone levels were evaluated in all participants included in the study. Vitamin D is hydroxylated in the liver to 25-hydroxy-vitamin D (25 (OH) D) which is widely used in clinical practice. The blood samples were drawn in the morning between 8 and 10 a.m. after an overnight fast of about 8-10 hr. Serum total testosterone level was verified using enzymatic methods with an autoanalyzer. The 25 (OH) D level was measured using high performance liquid chromatography method.

Continuous and categorical values were evaluated as frequency, mean ± SD or median and range as appropriate. Statistical analysis were performed using Student t test, Mann-Whitney U test and Chi-square test. Normality in the distribution of the data for each data was explored by Kolmogorov-Smirnov test. Any resulting significant association was included as continuous data in logistic regression to estimate if it could stand for a possible predictor for PE. Receiver operating characteristics (ROC) - derived areas under curve (AUC) were estimated to evaluate the diagnostic accuracy of vitamin D. The best cut-off value was determined using the Youden index. The sensitivity, specificity, positive and negative predictive values were compared by their 95% confidence intervals. Results were considered significant at p < 0.05. All statistical analysis were conducted using SPSS version 22 (IBM Co., Armonk, NY, USA).

## RESULTS

The participants in the present study (n = 161) were divided into 2 groups according to presence or absence of acquired PE. Acquired PE group contained 97 patients and control group had 64 men without PE. General demographic, sexual, and laboratory characteristics of the men with acquired PE and men in the control group are summarized in Table-1. The mean ages of the acquired PE group and control group were 40.0 ± 7.4 years and 40.5 ± 8.8 years, respectively (p = 0.939). There was also no significant difference between the groups regarding partner age, frequency of intercourse, BMI, smoking and alcohol use. Number of the individuals who had comorbidities, such as diabetes mellitus, hypertension, cardiovascular disease, and dyslipidemia were not significantly different between the groups.

**Table 1:** Demographic characteristics of study population.

Characteristics		Acquired PE (n = 97)		Control (n = 64)		p
		Mean ± SD	Median (range)	Mean ± SD	Median (range)	
		n - %		n - %		
Age (years)		40.0 ± 7.4	40 (21-63)	40.5 ± 8.8	40 (24-61)	0.939
Partner age (years)		36.4 ± 8.1	36 (20-47)	37.0 ± 8.2	37 (24-49)	0.253
BMI (kg/m^2^)		27.9 ± 4.3	28 (18.5-42)	27.4 ± 3.5	27.4 (19-38.2)	0.525
Frequency of Intercourse/ month		6.4 ± 4.2	6 (3-17)	7.1 ± 3.9	6 (2-18)	0.146
No. of smoker		42 – 43.3%		34 – 53.1%		0.222
No. of alcohol user		9 – 9.3%		11 – 17.2%		0.136
**Comorbidities**						
	DM	7 – 7.2 %		3 – 4.7%		0.515
	Hypertension	6 – 6.2%		6 – 9.4%		0.451
	CVD	2 – 2.1%		2 – 3.1%		0.671
	Dyslipidemia	3 – 3.1%		4 – 6.3%		0.336
IELT (seconds)		31.4 ± 23.4	25.2 (0-125)	213.7 ± 136.3	220 (85-850)	< 0.001
PEDT score		16.3 ± 3.8	17 (8-22)	9.7 ± 3.7	10 (2-15)	< 0.001
IIEF-5 score		21.4 ± 5.3	21 (16-24)	21 ± 4.0	21 (15-25)	1.000
BDI score		3.87 ± 3.32	4 (1-8)	3.50 ± 2.20	4 (2-9)	0.402
Vitamin D (ng/mL)		12.0 ± 4.5	11.4 (5.4-24.7)	18.2 ± 7.4	17 (11.6-46.3)	< 0.001
TT (ng/mL)		4.3 ± 1.7	4.1 (2.4-8.2)	4.5 ± 2.1	4.4 (2.6-7.9)	0.596

**PE** = premature ejaculation; **SD** = standard deviation; **BMI** = body mass index; **DM** = diabetes mellitus; **CVD** = cardiovascular disease; **IELT** = intravaginal ejaculatory latency time; **PEDT** = Premature Ejaculation Diagnostic Tool; **IIEF-5** = International Index of Erectile Function-5; **BDI** = Beck Depression Index; **TT** = total testosterone

The median PEDT scores were 17 (8-22) and 10 (2-15) in acquired PE group and control group, respectively (p < 0.001). The mean IELT were 31.4 ± 23.4 s and 213.7 ± 136.3 s in acquired PE group and control group, respectively (p < 0.001). Compared to men without PE, the patients with acquired PE had significantly lower 25 (OH) D levels (12.0 ± 4.5 ng/mL vs. 18.2 ± 7.4 ng/mL, p < 0.001). There was no statistically significant differences between the groups with respect to mean IIEF-5 score and mean total testosterone level.

**Figure 1:**
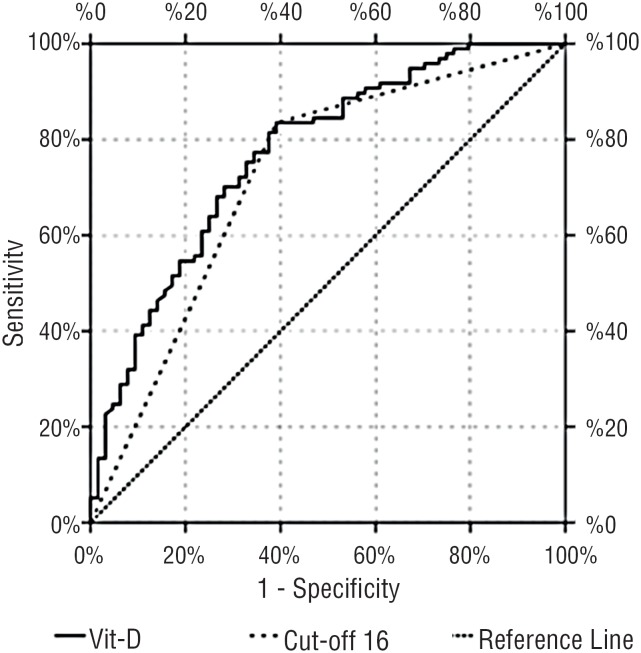
Receiver operating characteristic (ROC) curves of vitamin D for the prediction of premature ejaculation.

In the logistic regression analysis, vitamin D was found to be an independent risk factor for acquired PE, with estimated ORs (95% CI) of 0.639 (0.460-0.887, p = 0.007) (Table-2). The AUC of the ROC curve of vitamin D diagnosing acquired PE was 0.770 (95% CI: 0.695 to 0.844, p < 0.001) (Figure-1). The best cut-off value was defined for vitamin D, as determined by the Youden index to predict acquired PE. It was 16 ng/mL with a sensitivity of 60.9%, specificity of 83.5%, PPV of 70.9%, and NPV of 76.4% to indicate acquired PE (Table-3).

**Table 2:** Logistic regression analysis for premature ejaculation.

Univariable analysis			p	Multivariable analysis		p
	OR	95% CI		OR	95% CI	
IELT	0.912	0.878-0.948	< 0.001	0.893	0.843-0.947	< 0.001
PEDT score	1.544	1.354-1.760	< 0.001			
Vitamin D	0.827	0.770-0.888	< 0.001	0.639	0.460-0.887	0.007

**OR** = odds ratio; **CI** = confidence interval; **IELT** = intravaginal ejaculatory latency time; **PEDT** = Premature Ejaculation Diagnostic Tool

**Table 3:** The sensitivity, specificity, PPV, and NPV of vitamin D in patients with premature ejaculation.

	AUC	95% CI	p
Vitamin D	0.770	0.695-0.844	< 0.001
		Sensitivity	60.9%
		PPV	70.9%
		Specificity	83.5%
		NPV	76.4%

**AUC**=- area under curve; **CI** = confidence interval; **PPV** = positive predictive value; **NPV** = negative predictive value.

## DISCUSSION

The present study is the first study to research the relationship between acquired PE and serum vitamin D levels. In order to obtain satisfactory results from the treatments, diagnosis of the underlying etiological factors of acquired PE is very important. Effort to explain the etiology of acquired PE have comprised various range of theories. McMahon et al. reported in a review that acquired PE is prevalently due to anxiety, psychological problems, ED, prostatitis, hyperthyroidism, and recreational drugs (6). They also stated that men with acquired PE are usually older, have a higher BMI, and increased incidence of hypertension, diabetes mellitus, and ED compared to lifelong PE. Although in some patients the etiology of PE is not known it was demonstrated that among the four different PE categories, these comorbidities were more prevalent in acquired PE (14). In a recent study it was reported that a low serum level of vitamin D has a significant association with lifelong PE (33). However, lifelong PE suggested an underlying neurobiological functional discomfort; therefore, we included patients who had only ‘acquired’ PE which is more linked to underlying medical reasons.

Vitamin D deficiency has been related to a number of diseases such as, cardiovascular disease, hypertension, diabetes mellitus, obesity, several cancers, depression, and anxiety (19, 34). There is still a lack of consensus on the deficient and sufficient vitamin D value that varies from 20 ng/mL to 100 ng/mL in studies. However, there is a consensus that serum vitamin D levels below 10 ng/mL lead to risk of cardiovascular disease, cancer, and mortality (35). In the present study, the mean vitamin D levels were 12.0 ± 4.5 ng/mL and 18.2 ± 7.4 ng/mL in acquired PE group and control group, respectively. Although the sensitivity and specificity values are relatively low (60.9% and 83.5%, respectively), we think serum vitamin D levels should be investigate in the etiology of acquired PE.

The effect of vitamin D deficiency on PE is not clear. We think that vitamin D deficiency may affect PE with several mechanism. Firstly, several animal studies demonstrate that vitamin D deficiency resulted in anxiety-related behaviors (1921). Moreover, according to a case-control study, vitamin D deficiency seems more often in patients with anxiety (36). Anxiety has been known as a reason of PE by many authors (37, 38). It has been explained that anxiety activates the sympathetic nervous system and lowers the threshold for ejaculation (24). Gao et al. reported that higher rates of anxiety was found in acquired PE (39). Therefore, vitamin D deficiency-related anxiety might play an important role in underlying condition of acquired PE in some patients.

Ejaculation is controlled by complex reflex arc that includes afferent pathways, motor areas, efferent pathways, and ejaculatory organs, it is coordinated by multiple neural and muscular events (40). Blomberg Jensen et al. showed that the human epithelia of the ejaculatory tract are capable of metabolizing vitamin D and vitamin D receptors are concomitantly expressed in ejaculatory organs’ epithelia (41). Thus, vitamin D may indirectly but importantly affect the ejaculatory reflex arc (second possible mechanism).

The third possible mechanism underlying vitamin D - related PE is endocrinologic effect. Androgens play a crucial role in the regulation of ejaculation. Low or high serum testosterone levels have been incoherently associated with PE. Corona et al. demonstrated that delayed ejaculation is associated with lower testosterone level whereas a higher levels of testosterone characterizes PE (42). The data from the European Male Aging Study reported that vitamin D is positively associated with total testosterone (43). Wehr et al. has also showed that androgen levels were associated with vitamin D levels in a large study population (26). In our study, there was no statistically significant difference between the acquired PE group and control group with respect to mean total testosterone levels. Perhaps these results may contribute to clarify that the evidence on the relationship between vitamin D and testosterone levels in patients with PE seems insufficient.

The last mechanism may be related to nitric oxide (NO) and serotonin production. Low vitamin D level is associated with impaired production of NO and NO synthase (44). Therefore, vitamin D supplementation induces a significant increase in NO production (22). Patrick et al. reported that vitamin D controls the production of serotonin and Janssen et al. reported that NO and 5-hydroxytryptamine are important at the level of the sympathetic nervous system which can affect the ejaculation (23, 24). Thus, vitamin D may affect the ejaculatory control via NO production.

In the present study, the demographic data did not define significant association with acquired PE. Aging, smoking, alcohol consumption, comorbidities, such as diabetes, hypertension, cardiovascular disease did not reveal significant differences between the study groups. Gao et al. showed that patients with acquired PE had higher BMI (15). In our study, BMI did not identify significant association with the complaint of PE.

Several large observational studies reported that ED is more prevalent in patients with PE (1). However, there was no statistically significant difference between the groups with respect to mean IIEF-5 score, in our study. This may be the result of our study design which was an office-based research, so the participants of our study did not represent the general population and this is the first limitation of our study. Second, we did not study the free testosterone, luteinizing hormone, or prolactin levels to explain the evidence on the relationship between vitamin D and acquired PE. Third, the lack of anxiety assessing to clarify on the mechanism with respect to relationship between vitamin D and acquired PE. Fourth, partner status which can affect the ejaculation time was not evaluated and lastly, a wide range of exclusion criteria in this study may be a potential source of bias. Despite all these shortcomings, our study can be considered as an effort to reveal any relationship existing between vitamin D and acquired PE.

## CONCLUSIONS

Our research showed that men with acquired PE had lower levels of vitamin D compared with healthy men. We think that the evaluation of vitamin D levels may be used to explore the etiology and risk factors of acquired PE. However, this result is yet uncertain whether it could be provide a solution for acquired PE. Besides, patients with acquired PE should be questioned about vitamin D deficiency to avoid more serious problems. Future studies are required to confirm our results.

## ABBREVIATIONS

PE: Premature ejaculation
ISSM: International Society for Sexual Medicine
ED: Erectile dysfunction
NO: Nitric oxide
PEDT: Premature Ejaculation Diagnostic Tool IIEF-5 = International Index of Erectile Function-5
IELT: Intravaginal ejaculatory latency time
BDI: Beck Depression Inventory
BMI: Body mass index
25 (OH) D: 25-hydroxy-vitamin D
